# Correction: Mubarak et al. Enhanced Performance of Chitosan via a Novel Quaternary Magnetic Nanocomposite Chitosan/Grafted Halloysitenanotubes@ZnγFe_3_O_4_ for Uptake of Cr (III), Fe (III), and Mn (II) from Wastewater. *Polymers* 2021, *13*, 2714

**DOI:** 10.3390/polym17131759

**Published:** 2025-06-26

**Authors:** Mahmoud F. Mubarak, Ahmed H. Ragab, Rasha Hosny, Inas A. Ahmed, Hanan A. Ahmed, Salah M. El-Bahy, Abeer El Shahawy

**Affiliations:** 1 Petroleum Application Department, Egyptian Petroleum Research Institute (EPRI), Nasr City, Cairo 11727, Egypt; 2 Department of Chemistry, Faculty of Science, King Khalid University, Abha 62224, Saudi Arabia; ahrejab@kku.edu.sa (A.H.R.); eaahmed@kku.edu.sa (I.A.A.); 3 Production Department, Egyptian Petroleum Research Institute (EPRI), Nasr City, Cairo 11727, Egypt; rasha@epri.eg.org; 4 Petrochemicals Department, Egyptian Petroleum Research Institute (EPRI), Nasr City, Cairo 11727, Egypt; hanan@epri.eg.org; 5 Department of Chemistry, Turabah University College, Taif University, P.O. Box 11099, Taif 21944, Saudi Arabia; s.elbahy@tu.edu.sa; 6 Department of Civil Engineering, Faculty of Engineering, Suez Canal University, Ismailia 41522, Egypt

In the original publication, there was a mistake in Figures 5 and 8 as published [1].

The corrected version is displayed below.

3.1.3. X-Ray Diffraction (XRD)

The X-ray diffraction (XRD) patterns for chitosan, halloysite, and Zn@Fe_3_O_4_ material provide clear insights into their structural characteristics. The XRD pattern of chitosan shows distinct peaks at 2θ = 9.24° and 17.5°, which correspond to the (010) and (020) diffraction planes. These peaks confirm the semi-crystalline nature of chitosan, with broadening indicating the transformation from the crystalline structure of chitin to the more amorphous structure of chitosan. For halloysite, characteristic reflections at 2θ values of 11.08°, 21.42°, 27.45°, and 32.47° highlight its tubular structure and the crystallinity of this natural nanomaterial. Zn@Fe_3_O_4_ nanoparticles exhibit broad peaks at 2θ = 24.14°, 31.86°, 42.50°, 56.3°, and 61.787°, which are associated with the (210), (220), (400), (511), and (440) planes, confirming the formation of the ZnγFe_3_O_4_ phase, where zinc is doped into the magnetite (Fe_3_O_4_) structure. This confirms the formation of a well-integrated composite material with strong interactions between its components [38–40].

**Figure 5:**
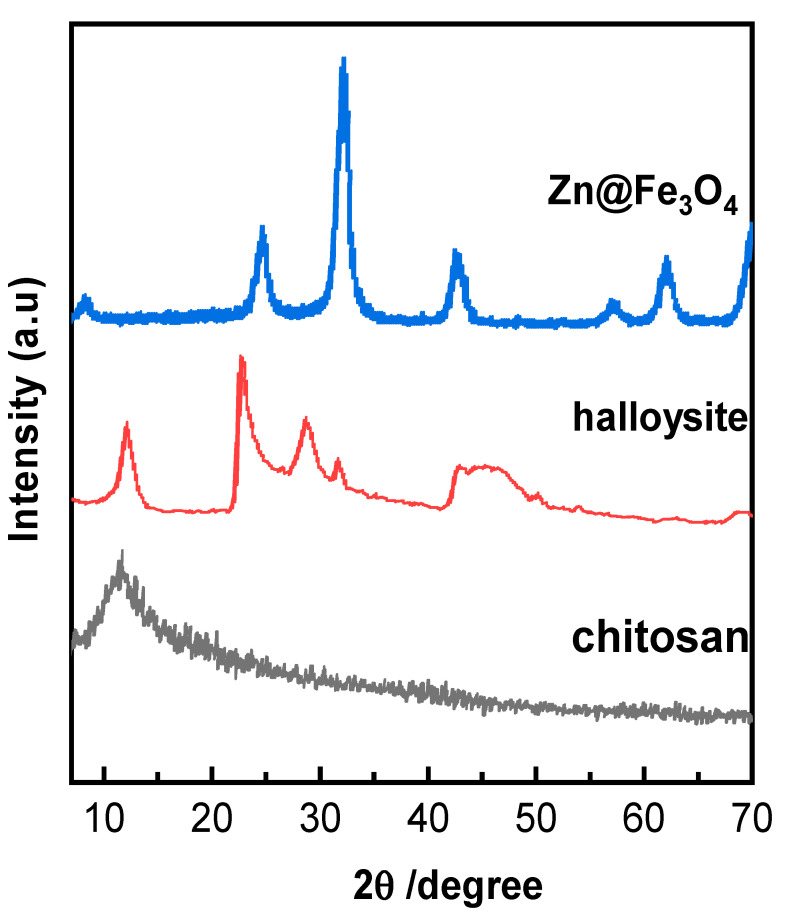
X-ray diffraction (XRD) patterns for chitosan, halloysite, Zn@Fe_3_O_4_, and composite.

3.1.7. TEM

Transmission electron microscopy (TEM) was used for the morphological study of loaded and discharged ZnγFe_3_O_4_ halloysite tubes. The TEM image shows halloysite nanotubes as elongated, cylindrical rods, with diameters ranging between 40 and 80 nm and lengths varying from 500 to 1000 nm (Figure 8). The nanotubes are surrounded by clusters of smaller particles, possibly indicating surface interactions or modifications. The TEM images of the loaded halloysite ZnFe_3_O_4_ have a morphology, validated by TEM analysis, that is random and free of any defects (i.e., beads, globules, and undefined form). It can be concluded that the ZnγFe_3_O_4_-loaded halloysite has similar morphological characteristics (Figure 8). ZnγFe_3_O_4_ was filled with average diameters of 150 ± 45 nm.

**Figure 8:**
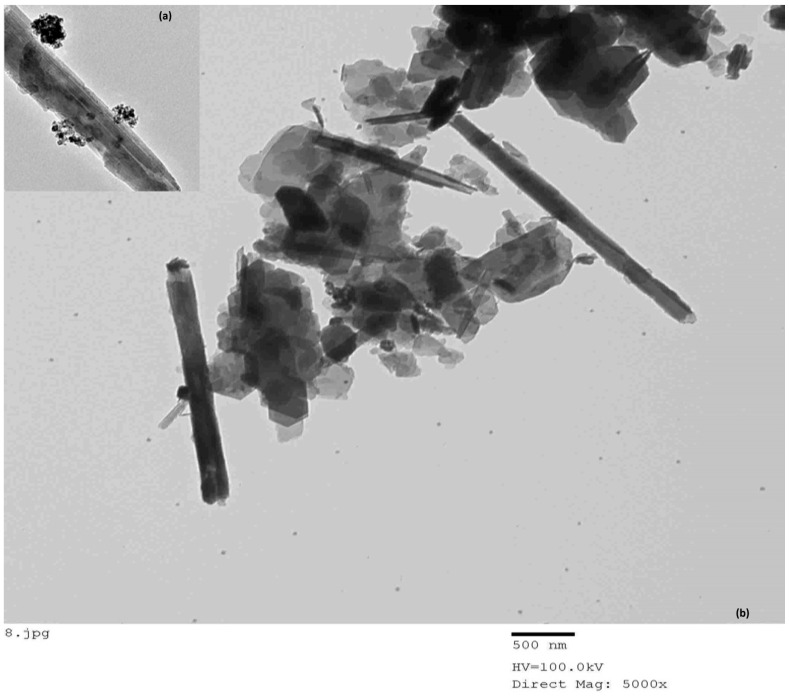
TEM of (**a**) halloysite and (**b**) magnetic halloysite.

The authors state that the scientific conclusions are unaffected. This correction was approved by the Academic Editor. The original publication has also been updated.
