# Supplementary material for: Enhanced Performance of Chitosan via a Novel Quaternary Magnetic Nanocomposite Chitosan/Grafted Halloysitenanotubes@ZnγFe3O4 for Uptake of Cr (III), Fe (III), and Mn (II) from Wastewater
Source: Polymers (Basel). 2021 Aug 13;13(16):2714. doi: 10.3390/polym13162714 (PMC8398450; doi:10.3390/polym13162714)
Supplement: Supplementary file 1 [file polymers-13-02714-s001.zip › polymers-1324247-supplementary.pdf]

Supplementary Materials

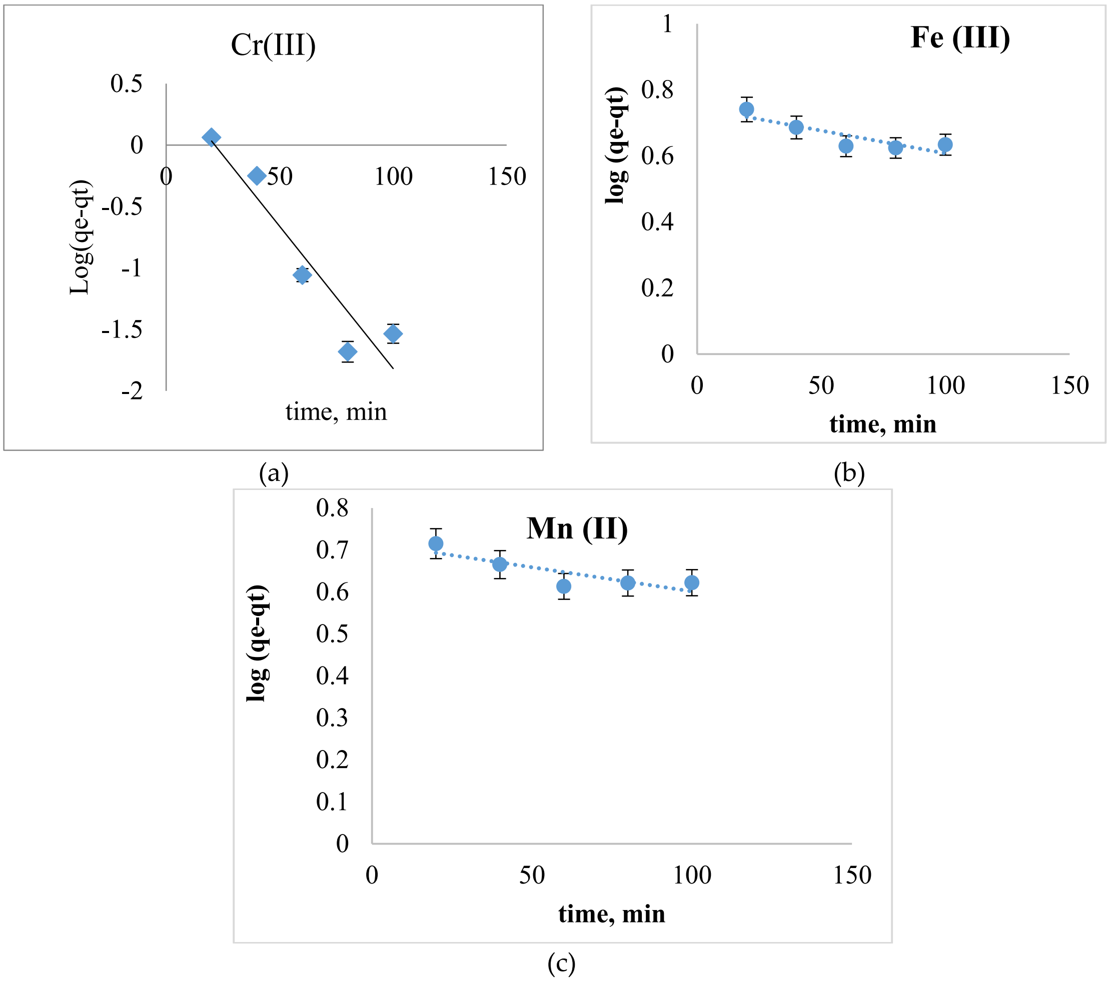

Figure S1. Pseudo-first-order rate (60 mg/L) for (**a**) Cr (III), (**b**) Fe (III), and (**c**) Mn (II) removal on/g-HNTs@ZnγM adsorbent.

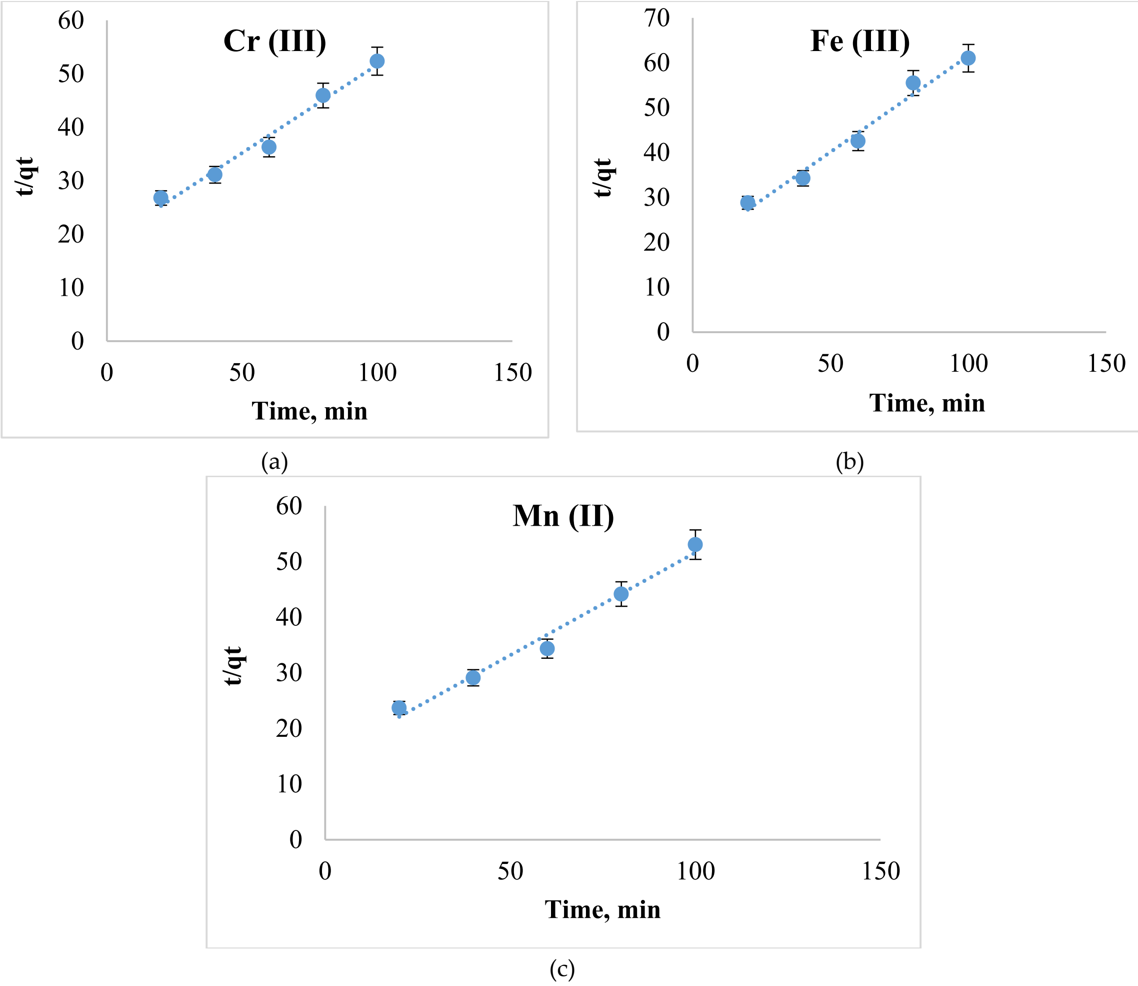

Figure S2. Pseudo second-order model (60 mg/L) for (**a**) Cr (III), (**b**) Fe (III), and (**c**) Mn (II) removal on Ch/g-HNTs@ZnγM adsorbent.

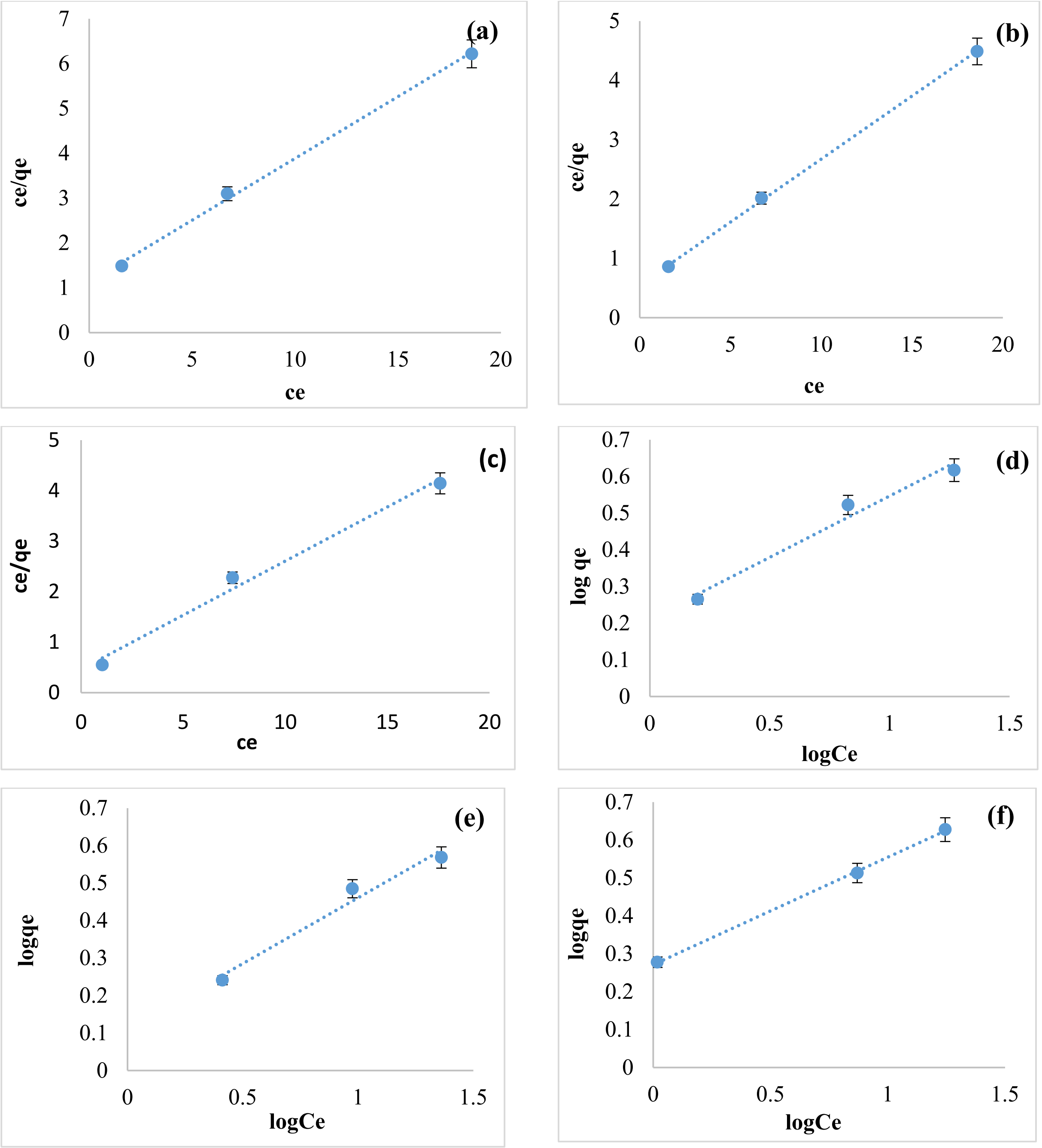

Figure S3. Langmuir model for (**a**) Cr (III), (**b**) Fe (III),and(**c**) Mn (II) and Freundlich model for (**d**) Cr (III), (**e**) Fe (III), and (**f**) Mn (II).

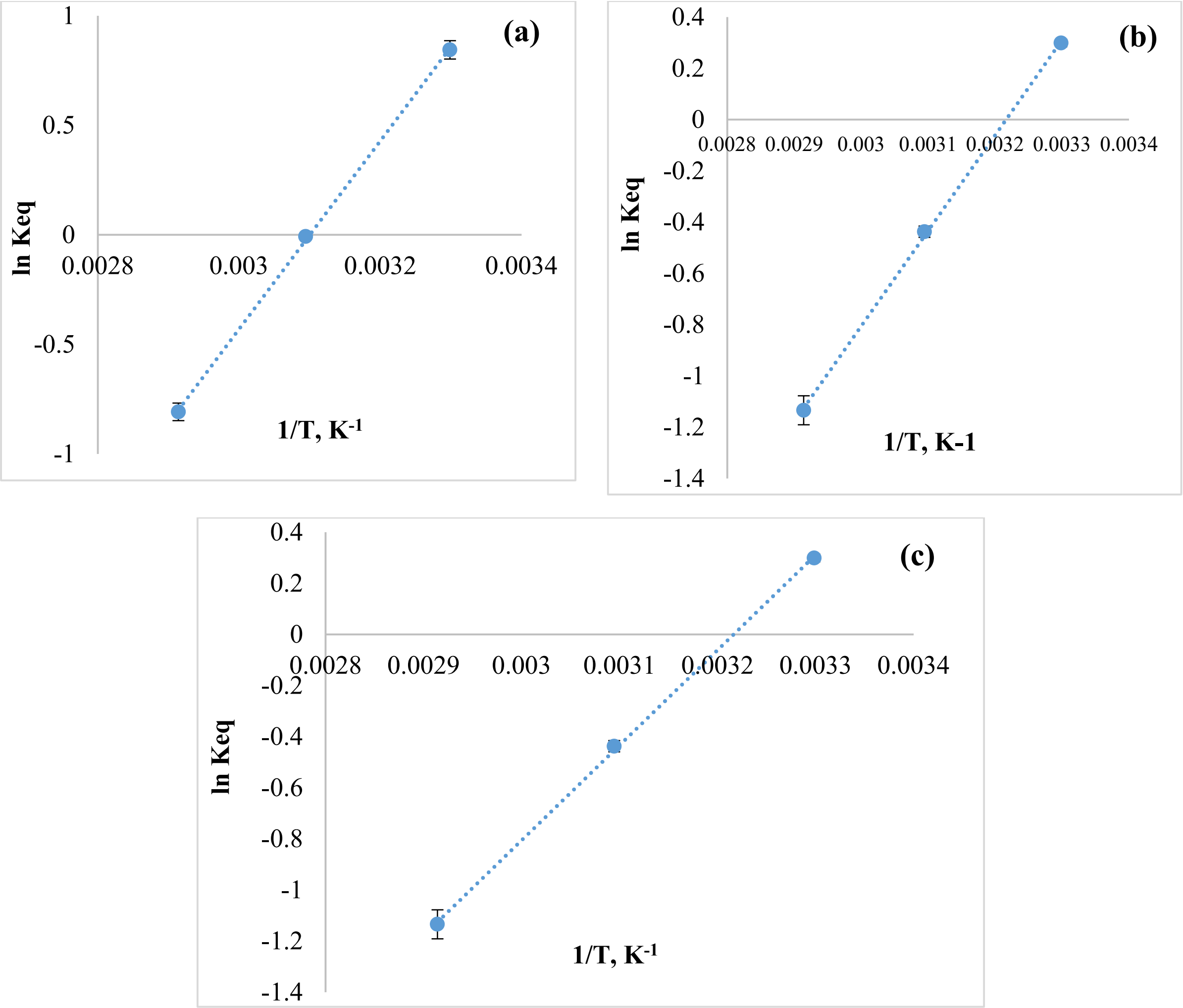

Figure S4 Thermodynamic adsorption parameters for the adsorption of (**a**) Cr (III), (**b**) Fe (III), and (**c**) Mn (II) on Ch/g-HNTs@ZnγM.
